# Spatiotemporal patterns of drug use disorder in Sweden assessed using population-based registries

**DOI:** 10.1186/s12889-023-15149-1

**Published:** 2023-02-07

**Authors:** Kathleen Stewart, Kenneth S. Kendler, Anton Westholm, Henrik Ohlsson, Jan Sundquist, Kristina Sundquist

**Affiliations:** 1 Center for Geospatial Information Science, Department of Geographical Sciences, University of Maryland, College Park, MD 20742 USA; 2 Virginia Institute for Psychiatric and Behavioral Genetics, Virginia Commonwealth University, Richmond, VA USA; 3 Department of Psychiatry, Virginia Commonwealth University, Richmond, VA USA; 4 Center for Primary Health Care Research, Lund University, Malmö, Sweden; 5 Department of Family Medicine and Community Health, Icahn School of Medicine at Mount Sinai, New York, NY USA; 6 Department of Population Health Science and Policy, Icahn School of Medicine at Mount Sinai, New York, NY USA

**Keywords:** Drug use disorder, Substance use, Spatiotemporal clustering, SaTScan, Geographic patterns

## Abstract

**Background:**

Drug Use Disorder (DUD) is a major contributor to world-wide morbidity and mortality. The extensive national registers in Sweden provide the basis for a study of spatial and temporal patterns of DUD onset and recurrence in Sweden from 2001–2015.

**Methods:**

To identify patterns of DUD over space, time and gender for Swedish individuals aged 15–35, space–time clustering using SaTScan was applied. We used yearly information on residential locations in Demographic Statistical Areas (DeSO) for all of Sweden. The clustering analysis used a Poisson probability model and a null hypothesis that the expected number of cases in each DeSO was proportional to the population size of DeSOs. As SaTScan results can be unstable, steps were taken to determine stable clusters and to refine and optimize cluster size. Results for each gender-register combination were compared to the results of spatial clustering using Gi* statistics. The space–time scanning model was also run with an adjustment for neighborhood socioeconomic status to determine DUD prevalence as it relates to education, income, unemployment and receipt of social welfare.

**Results:**

DUD prevalence increased over time. Males yielded more significant clusters than females for both criminal and medical registers. Female DUD prevalence rates increased over time, especially after 2012. Higher correlations in DUD rates existed across the two registers than across gender. Male clusters were present from 2004 onwards while female–criminal clusters appeared after 2007, and female–medical clusters not until 2010. By 2013, clusters existed for all gender–register combinations. Male–criminal clusters were concentrated in Stockholm, Göteborg and Malmö as were male and female-medical clusters. Neighborhood SES was more highly related to the distribution of criminal than medical DUD clusters. A persistent gap in core clusters was identified in Stockholm in an area with notably high SES.

**Conclusions:**

Persistent hotspots of DUD in Sweden were confirmed as well as new and emerging hotspots, especially in Stockholm, Göteborg and Malmö. Higher correlations existed in DUD rates across registers than across gender. The findings are useful for monitoring the current drug problem and for identifying drivers underlying patterns of spread and important causal pathways to DUD.

## Background

Drug Use Disorder (DUD), a major contributor to both morbidity [1] and mortality [2–4] in both the United States and in many other countries, is a critical public health concern. The substantial research on the etiology of DUD suggests that it is a classical multifactorial disorder with a wide array of risk factors from several key domains especially biological/genetic, psychological/developmental and socio-cultural [5]. In addition to these traditional risk factor domains, extensive evidence suggests that DUD clusters in both space and time [6–10]. Detailed studies of such clusters provide the opportunity to delineate further potentially important causal pathways to DUD.

Fewer studies, however, have explored space–time clustering of DUD on a national scale. The high quality medical and criminal registers in Sweden are among the best in the world for the breadth and quality of coverage and make such a national study feasible. These registers include the recently created primary care register [11], and contain nationwide annually-updated information about the residential locations of all individuals aggregated to Demographic Statistical Area (DeSO) level (see below for details). They have been used previously to examine both the impact of genetic risk [12, 13] and community environments [13–16] on risk for DUD. Of particular relevance, prior work has shown that combined household/community effects accounted for ~ 8% of the total population variation in DUD in Sweden [17] and sewage water from 33 different municipalities across Sweden support substantial geographical differences in illicit drug consumption suggesting considerable variation in illicit drug consumption across regions of Sweden [18].

In this first examination of the national distribution of DUD in the country of Sweden, we ask the following major questions:

1. Can we identify patterns of onset and recurrence of DUD over space and time at national level, across Sweden?
2. How similar are the identified clusters in men and women and in those whose DUD is ascertained from criminal *versus* medical registers?
3. To what extent does the observed clustering of DUD overlap with geographical clusters of socioeconomic deprivation?

This study examines national patterns of DUD in Sweden between 2001 and 2015 for ages 15–35 years. We use spatiotemporal clustering to reveal in detail how DUD, by gender and register, has varied across space and with a particular focus on where DUD occurrences have emerged, persisted, or possibly increased over time. These patterns of change are critical to not only improving our understanding of causal pathways but also providing public health decision makers and stakeholders with information to guide treatment service and mitigation planning.

## Methods

### Data and study area

This study used data on individuals from Swedish population-based registers with national coverage linking each person’s unique personal identification number, which, for confidentiality, was replaced with a serial number by Statistics Sweden. Our dataset consisted of individuals residing in Sweden for the years 2001 through 2015. For each year, we selected individuals aged 15 through 35 when a first registration for DUD is most likely to occur [19]. In this dataset, we included the date of registration for DUD. DUD was defined based on registration either in the medical registers (the Inpatient Register and Specialist Care Register) or the criminal registers (the Swedish Conviction and Suspicion Registers). In the medical registers, DUD was identified by ICD-10-codes for mental and behavioral disorders due to psychoactive substance use (F10-F19), except those due to alcohol (F10) or tobacco (F17). In the criminal registers, DUD was defined by codes 3070, 5010, 5011, and 5012 in the Suspicion register, and in the Conviction register by references to laws covering narcotics (law 1968:64, paragraph 1, point 6) and drug-related driving offenses (law 1951:649, paragraph 4, subsection 2 and paragraph 4A, subsection 2). For our analysis, we separated the medical and criminal registrations so that an individual could have both types of registrations during a year. We created 15-yearly subsets for individuals aged 15 to 35 for a given year, and for this analysis, we counted the number of individuals with both types of registrations relating to DUD for a given year.

We also included information on the area of residence of each individual by year. We used “Demografiska Statistikområden” (DeSO) (i.e., Demographic Statistical Areas). Historically, studies on neighborhood effects in Sweden have tended to use the older Small Areas for Market Statistics (SAMS) zone design [20]. DeSOs are also a zone design, implemented by Statistics Sweden (SCB) in 2018 as a new and modernized successor to SAMS [21]. DeSOs subdivide Sweden into 5,984 areas using a target population size per area of 1,500 people [21]. As the design is based on population size, the geographic size of a DeSO varies with population density. The divisions between zones are designed to follow barriers such as water bodies, railroads etc., in order to capture neighborhood interactions, and the zone design is constructed with special consideration for the analysis of neighborhood effects and the spatial dimensions of socio-economy [22]. The spatial data for DeSOs used in this research was version v2 acquired from Statistics Sweden [23].

## DUD prevalence across space and time, significance and reliability

To detect patterns of DUD at DeSO-level, a space–time clustering analysis was undertaken using SaTScan version 9.7 [24]. SaTScan was developed for the purpose of finding statistically significant clusters across space and time [25] and has been applied with modifications, for example, refining and optimizing cluster size [26, 27]. In this study, the SaTScan analysis was undertaken for DUD case counts in both the criminal and medical registers, where a case represents any individual who has had at least one DUD registration (either criminal or medical) during the relevant year. The clustering approach used a Poisson probability model that scanned for areas with high numbers of DUD cases using a null hypothesis that the expected number of cases in each DeSO was proportional to the population size of the DeSO [25]. SaTScan tests that there is no elevated risk within each scanning window against the alternative hypothesis that elevated risk is present. The likelihood function used a Poisson assumption for a given scanning window and followed the approach used by SaTScan [24]. This likelihood function is maximized over all scanning windows by DeSO and year, with the window that has the highest likelihood ratio being considered as the most likely cluster. Remaining clusters are considered secondary and are ordered by the same likelihood ratio.

As SaTScan results can be relatively unstable, yielding different results depending on the chosen scanning window, Chen et al. [26] used a *reliability index* in order to find stable clusters and other studies have also applied a similar index [8]. We computed this index by first running SaTScan multiple times with different population windows and counting the number of instances where a given location determines a set of stable clusters. The ratio produced by the number of times a DeSO appeared in these clusters over the total number of scans constituted the reliability index *R,* where *N* refers to the number of scans and *I* refers to the number of times the DeSO appears in a core cluster in any of the scans [26].

$${R}_{i}=\frac{{I}_{i}}{N}$$

To determine the range for our scanning windows, one-year prevalence rates of DUD were analyzed for all combinations of gender and register types (i.e., female-medical, female-crime, male-medical, male-crime) producing a range of prevalence from 0.2% to 2.7%. Based on this finding, SaTScan was run from a 0.1% to a 3% sized population window using 0.1% intervals, as well as at 4% and 5%, for each gender and register combination.

SaTScan also supports the computation of relative risk for each location [24]. A *core cluster* was defined as a cluster where a minimum of two-thirds (66%) of the spatial enumeration units had a relative risk of 1.5 or more [8]. We refined this notion of core cluster further by applying a Bonferroni test [28] using a confidence level of 0.01, and dividing by the total number of scans. As both the registers and population data were aggregated by year, the scanning time window was limited to 1 year for all scans.

As further validation of the extent that spatial clustering of DUD rates for this age group was present for the different registers by gender throughout the time period, we also computed Gi* statistics, implemented using the Python Spatial Abstraction Library. This statistic was calculated yearly for each gender-register combination using a symmetrically-weighted queen contiguity spatial weights matrix for all DeSO areas. The Gi* statistic was computed for one-year prevalence rates by DeSO, correcting the rate for the number of individuals within each DeSO. This was done by multiplying the rates with a shrinkage factor (SF) used in multilevel models. This produces more shrinkage if the number of individuals in the DeSO area is small [29]. These results were compared to the SaTScan clustering results to provide some quality assurance for the space–time clustering results.

Community measures of neighborhood deprivation have typically predicted future risk for DUD in the US [30] and in Sweden where follow-up analyses suggest that the association is likely to be largely causal in nature [15]. In order to ascertain the relationship between core clusters and neighborhood socioeconomic status (SES), we also ran the model with an adjustment for SES. For each DeSO zone and year, a neighborhood SES index was created based on register data for all residents in a DeSO aged 25–64 representing the age range that are the most socioeconomically active [31]. Neighborhood SES was based on four items: low education level (years of formal education), low income (from all sources, including interest and dividends; defined as income below 50% of median individual income), unemployment (excluding full-time students, those completing military service, and early retirees), and receipt of social welfare [31]. For this research, the index was used to categorize neighborhood SES into three classes, low (more than one standard deviation below the mean), moderate (within one SD of the mean), and high (more than one SD above the mean). All DeSO areas were classified into one of these three classes of neighborhood SES (low, medium and high), analyzed as a categorical covariate using SaTScan, and mapped as neighborhood SES values, -1 through 1.

## Results

The population was stratified by gender, type of register, and the combination of both register and gender (Table 1). Prevalence was measured as the ratio of the number of individuals with at least one DUD-registration in each register by the population residing in each geographic unit during each year. Three trends in this table are noteworthy. First, the prevalence of DUD was increasing relatively steadily over this time period. Second, males were much more likely to be registered for DUD than females. Third, the male to female prevalence ratio for DUD was consistently higher in the criminal than in the medical register.

**Table 1:** Population aged 15 through 35 years for 2001 through 2015 by gender, type of register, and the combination of both gender and register

Year	Total Population	Female Population (%)	Male Population (%)	Total DUD Cases	Female DUD Cases	Male DUD Cases	Total Crime DUD Cases	Female Crime DUD Cases (%)	Male Crime DUD Cases (%)	Total Medical DUD Cases	Female Medical DUD Cases (%)	Male Medical DUD Cases (%)
2001	2,364,138	49.0%	51.0%	11,967	2817	9150	8530	16.3%	83.7%	4695	36.4%	63.6%
2002	2,357,408	49.0%	51.0%	13,466	3095	10,371	9917	16.0%	84.0%	4988	36.7%	63.3%
2003	2,358,738	49.0%	51.0%	13,726	3055	10,671	10,321	15.4%	84.6%	4817	37.0%	63.0%
2004	2,370,157	49.0%	51.0%	15,634	3394	12,240	11,711	15.2%	84.8%	5665	35.1%	64.9%
2005	2,396,365	49.0%	51.0%	17,475	3620	13,855	13,492	15.2%	84.8%	5892	34.1%	65.9%
2006	2,430,637	48.9%	51.1%	20,436	4109	16,327	15,992	14.9%	85.1%	7033	33.0%	67.0%
2007	2,461,545	48.8%	51.2%	23,231	4711	18,520	18,228	14.8%	85.2%	7864	33.6%	66.4%
2008	2,488,347	48.8%	51.2%	25,973	5308	20,665	20,323	14.8%	85.2%	8962	33.9%	66.1%
2009	2,517,503	48.8%	51.2%	28,607	5538	23,069	22,142	13.7%	86.3%	10,118	32.5%	67.5%
2010	2,532,955	48.8%	51.2%	31,036	5901	25,135	24,380	13.7%	86.3%	10,764	31.7%	68.3%
2011	2,536,839	48.8%	51.2%	32,760	6133	26,627	25,705	13.1%	86.9%	11,231	32.0%	68.0%
2012	2,544,155	48.8%	51.2%	35,298	6571	28,727	27,445	13.2%	86.8%	12,265	31.3%	68.7%
2013	2,557,162	48.8%	51.2%	36,364	6940	29,424	27,590	13.4%	86.6%	13,393	31.1%	68.9%
2014	2,578,920	48.7%	51.3%	36,321	7153	29,168	27,014	13.8%	86.2%	14,393	30.8%	69.2%
2015	2,600,513	48.6%	51.4%	36,620	7496	29,124	26,373	13.8%	86.2%	14,991	32.2%	67.8%

To obtain an overview of the stability of rates of DUD registration across time, gender and both medical and criminal registers, we calculated the Pearson product moment correlation matrices using data from 2001–2015, by registration and by sex. Across these years, the correlation between medical and criminal registrations was substantial (mean r =  + 0.52) (Table 2) while the correlation between genders was more modest (mean r =  + 0.21) (Table 3). The temporal stability was somewhat higher in the criminal than medical register for males. The correlations across time were moderate for both register and gender, and tended to decline with time. This analysis helped guide our expectations for both the spatial and space–time analysis as will be described in detail in the following sections.

**Table 2:** Pearson correlation values for rates across DeSO areas (2001 to 2015) by Type of Registration

	MEDICAL ABOVE DIAGONAL AND CRIMINAL BELOW															
	**2001**	**2002**	**2003**	**2004**	**2005**	**2006**	**2007**	**2008**	**2009**	**2010**	**2011**	**2012**	**2013**	**2014**	**2015**	Medical with Criminal
**2001**	1.00	0.31	0.19	0.34	0.14	0.17	0.28	0.21	0.16	0.08	0.23	0.11	0.12	0.23	0.23	0.38
**2002**	0.47	1.00	0.22	0.41	0.29	0.25	0.23	0.13	0.09	0.19	0.14	0.12	0.08	0.09	0.14	0.56
**2003**	0.31	0.26	1.00	0.31	0.39	0.22	0.27	0.11	0.08	0.11	0.23	0.16	0.20	0.16	0.16	0.50
**2004**	0.38	0.36	0.44	1.00	0.34	0.44	0.21	0.22	0.29	0.11	0.18	0.18	0.17	0.16	0.30	0.54
**2005**	0.21	0.16	0.44	0.60	1.00	0.41	0.31	0.24	0.19	0.27	0.20	0.26	0.12	0.26	0.29	0.59
**2006**	0.29	0.19	0.37	0.45	0.50	1.00	0.50	0.25	0.13	0.15	0.17	0.19	0.22	0.30	0.35	0.64
**2007**	0.24	0.21	0.42	0.38	0.37	0.59	1.00	0.32	0.28	0.17	0.28	0.19	0.13	0.26	0.21	0.66
**2008**	0.27	0.24	0.29	0.32	0.34	0.40	0.54	1.00	0.49	0.48	0.35	0.31	0.15	0.32	0.21	0.61
**2009**	0.22	0.30	0.21	0.33	0.22	0.20	0.35	0.50	1.00	0.48	0.30	0.28	0.18	0.24	0.17	0.25
**2010**	0.22	0.25	0.17	0.20	0.16	0.28	0.34	0.50	0.37	1.00	0.29	0.42	0.16	0.24	0.31	0.70
**2011**	0.29	0.31	0.24	0.23	0.23	0.27	0.23	0.34	0.30	0.44	1.00	0.46	0.24	0.31	0.48	0.53
**2012**	0.23	0.24	0.24	0.29	0.21	0.25	0.27	0.29	0.42	0.27	0.41	1.00	0.35	0.42	0.42	0.45
**2013**	0.28	0.14	0.23	0.29	0.37	0.28	0.30	0.34	0.26	0.37	0.39	0.45	1.00	0.49	0.40	0.44
**2014**	0.30	0.26	0.36	0.30	0.34	0.32	0.38	0.46	0.38	0.32	0.41	0.38	0.43	1.00	0.46	0.70
**2015**	0.25	0.19	0.32	0.31	0.30	0.26	0.37	0.41	0.32	0.30	0.35	0.44	0.40	0.59	1.00	0.59

**Table 3:** Pearson correlation values for rates across DeSO areas (years 2001 to 2015) by Sex

	FEMALES ABOVE DIAGONAL AND MALES BELOW															
	**2001**	**2002**	**2003**	**2004**	**2005**	**2006**	**2007**	**2008**	**2009**	**2010**	**2011**	**2012**	**2013**	**2014**	**2015**	Males with Females
**2001**	1.00	0.30	0.16	0.06	0.10	0.08	0.28	0.29	0.20	0.06	0.08	0.07	0.02	0.12	0.27	0.25
**2002**	0.48	1.00	0.25	0.32	0.32	0.31	0.16	0.09	0.07	0.18	0.04	0.11	0.05	0.13	0.15	0.31
**2003**	0.33	0.34	1.00	0.25	0.35	0.18	0.33	0.09	0.05	0.13	0.08	0.06	0.02	0.14	0.20	0.23
**2004**	0.47	0.44	0.54	1.00	0.46	0.31	0.10	0.37	0.20	0.18	0.08	0.17	0.11	0.16	0.06	0.26
**2005**	0.23	0.25	0.52	0.53	1.00	0.36	0.26	0.28	0.21	0.17	0.11	0.07	0.05	0.05	0.11	0.22
**2006**	0.27	0.32	0.46	0.46	0.58	1.00	0.20	0.12	0.08	0.13	0.11	0.10	0.05	0.14	0.24	0.22
**2007**	0.34	0.34	0.42	0.41	0.46	0.68	1.00	0.65	0.23	0.27	0.18	0.22	0.08	0.29	0.21	0.29
**2008**	0.34	0.23	0.34	0.32	0.39	0.40	0.44	1.00	0.53	0.28	0.09	0.10	0.07	0.36	0.13	0.26
**2009**	0.34	0.33	0.31	0.44	0.40	0.36	0.40	0.61	1.00	0.37	0.10	0.06	0.17	0.13	0.17	0.11
**2010**	0.26	0.25	0.23	0.23	0.23	0.27	0.33	0.51	0.52	1.00	0.34	0.17	0.12	0.19	0.09	0.29
**2011**	0.36	0.34	0.33	0.32	0.33	0.32	0.31	0.39	0.42	0.50	1.00	0.53	0.17	0.14	0.19	0.09
**2012**	0.28	0.26	0.30	0.36	0.30	0.34	0.34	0.36	0.41	0.36	0.55	1.00	0.56	0.32	0.21	0.18
**2013**	0.32	0.19	0.37	0.38	0.39	0.39	0.37	0.33	0.41	0.38	0.38	0.51	1.00	0.45	0.35	0.20
**2014**	0.38	0.24	0.41	0.38	0.45	0.43	0.40	0.37	0.45	0.40	0.47	0.55	0.65	1.00	0.43	0.33
**2015**	0.37	0.27	0.39	0.39	0.39	0.34	0.42	0.34	0.33	0.37	0.50	0.48	0.44	0.59	1.00	0.33

## DUD cluster patterns by gender and register

Across all scans, males in this 15–35-year age group yielded around twice the number of significant clusters (P < 0.05) compared to females, for both criminal and medical registers. After core clusters had been computed, this disparity increased further such that the number of male DUD core clusters associated with the criminal register were more than eight times higher than the number of female core clusters for this same register, and more than twice the number of female core clusters for the medical register (Table 4) These figures parallel the gender differences in the number of DUD cases from the criminal and medical registers.

**Table 4:** Cluster counts by gender and register

	**Criminal**	**Medical**
*Significant clusters (P* < = *0.05)*		
Male	2844	2145
Female	1430	1289
*Core clusters*		
Male	1396	679
Female	160	262

**Fig. 1:**
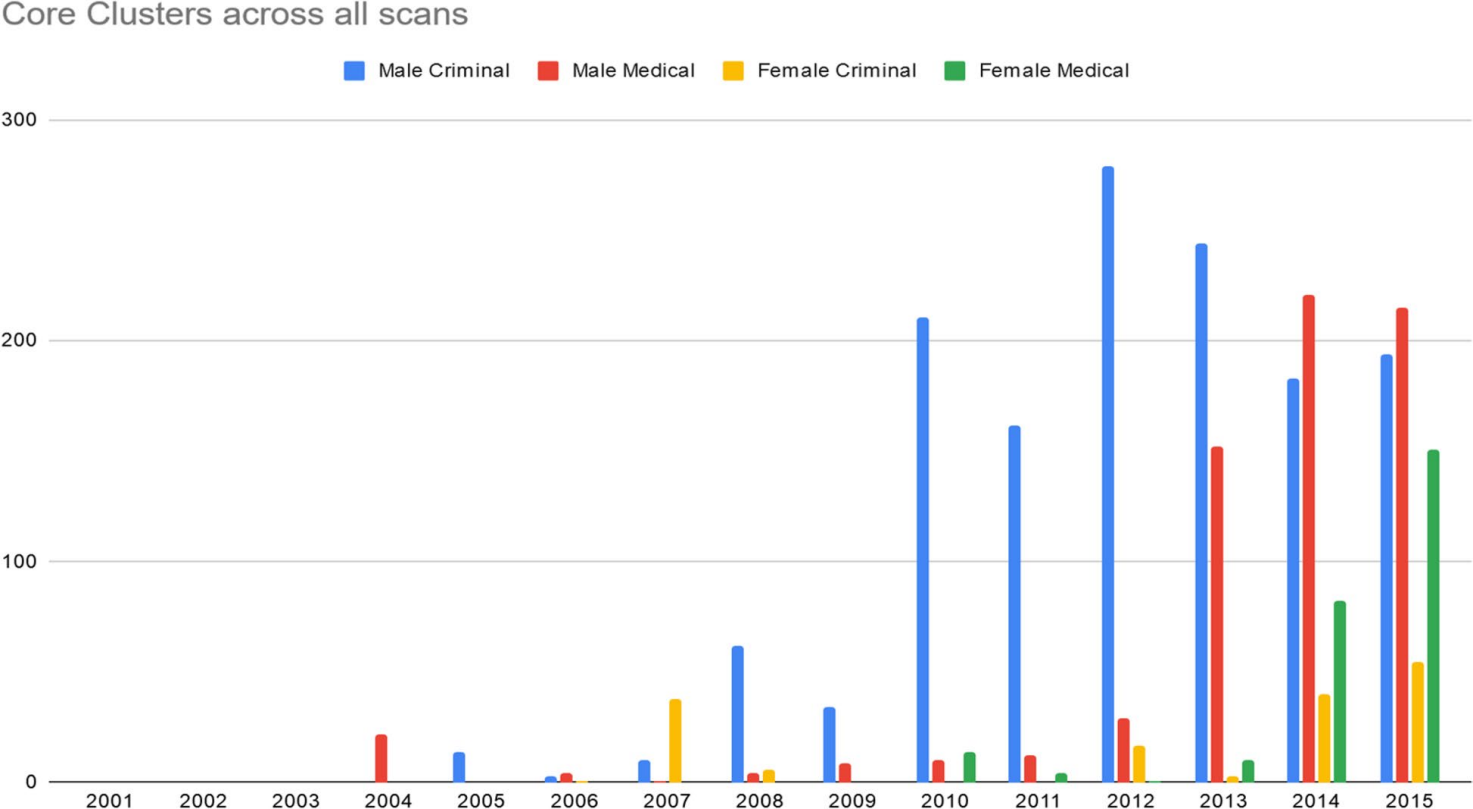
Number of core clusters across all scans for combinations of gender and register

Over the study time period, core clusters capturing male DUD prevalence associated with the criminal register emerged in 2004 with a notable increase from 2010 to 2015 (Fig. 1). Core clusters for male–medical register cases appeared in 2005 with a noticeable spike in 2013. Female–criminal core clusters were first noted in 2007, while female–medical clusters appeared later than for males in 2010, and were less temporally concentrated overall while being skewed toward the last half of the study period. Results showed that by 2013 and for the last three years of the study period, core clusters for *all* gender–register combinations could be found.

## DUD rates across Sweden

**Fig. 2:**
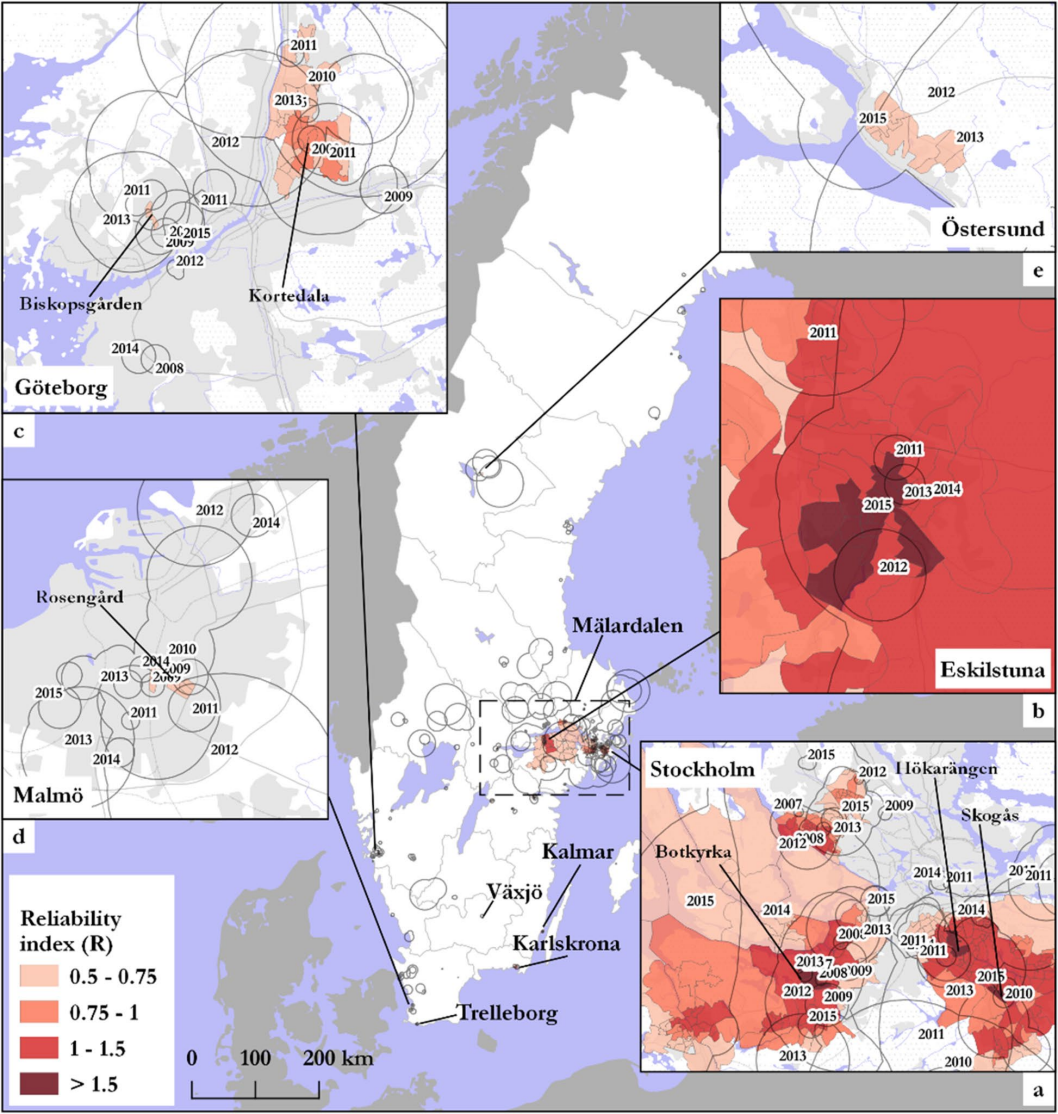
Spatial pattern of core clusters across Sweden. To reduce detail in this map, the boundaries of overlapping clusters from the same year have been dissolved

At the national level, the overall pattern saw a concentration of DUD prevalence in and around the capital, Stockholm. Within Stockholm County, DeSOs with reliably high DUD prevalence (high *R*_*i*_) were found in the southern suburbs centered around Hökarängen, Botkyrka and Skogås (Fig. 2, inset map a). DeSOs with reliably high DUD prevalence extended out from the city center to the mid-sized city of Eskilstuna, just west of Stockholm (Fig. 2, inset map b). Sweden’s second largest city, Göteborg, also had similar areas of high DUD prevalence in the northern suburbs, mainly around Biskopsgärden and Kortedala (Fig. 2, inset map c). Sweden's third largest city, Malmö, saw similarly high DUD prevalence, but clusters there were less persistent over time yielding lower reliability scores than either Stockholm and Göteborg (Fig. 2, inset map d).

Outside of Mälardalen and Göteborg, locations with DeSOs with reliably high DUD prevalence (*R*_*i*_ > 0.75) were found in the South (Karlskona, Trelleborg, Växjö, and Kalmar) (Fig. 2, main map) and Östersund in the north (Fig. 2, inset map e). No areas with a *R*_*i*_ above 0.75 were found north of Stockholm, except for Östersund (Fig. 2, inset map e), although clusters of DUD prevalence were found in certain northern cities including Sundsvall, Umeå and Luleå (Fig. 2 main map).

## Spatial patterns of DUD prevalence by gender and register

**Fig. 3:**
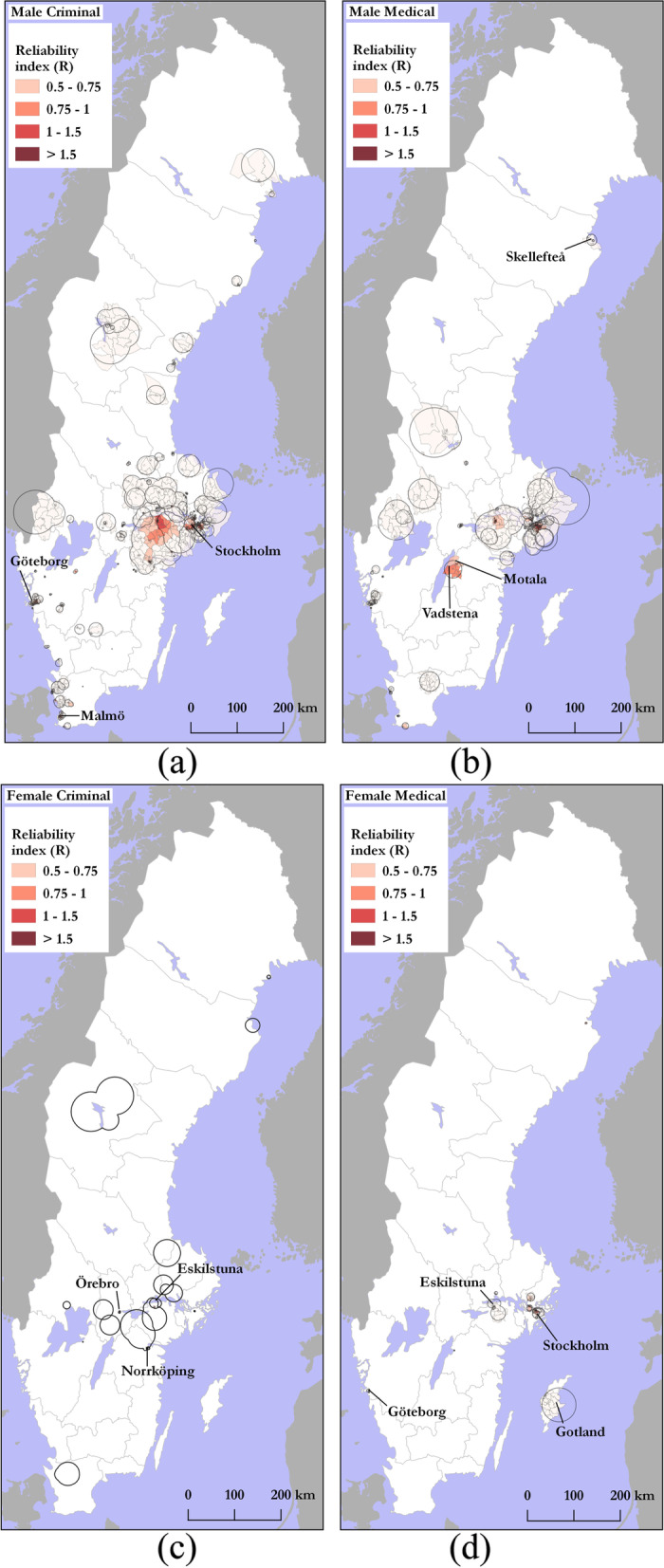
Core cluster locations across gender-register categories: **a** male–criminal (**b**) male–medical, **c** female–criminal, and (**d**) female–medical. Cluster geometries dissolved by year

The pattern of core clusters for male–criminal DUD for the age group 15–35 years was similar to the overall pattern seen in Fig. 2, with higher DUD prevalence and a concentration of clusters in the cities of Stockholm, Göteborg and Malmö, as well as in cities along the northeast coast and Östersund (Fig. 3a). For male–medical DUD prevalence, the pattern was similar, but core clusters were also found in the area of Vadstena and Motala (south central Sweden) (Fig. 3b). There were no core clusters for this group in any northern city with the exception of Skellefteå (Fig. 3b). For female–criminal rates, there were generally fewer core clusters, however, higher DUD prevalence was found west and south of Stockholm in cities, such as Örebro, Eskilstuna and Norrköping, while any concentration around Stockholm was absent (Fig. 3c). Locations on the west coast of Sweden that were found for males, were not present for females. Higher prevalence of female–medical DUD rates were returned for DeSOs around Stockholm, Göteborg and Eskilstuna, with core clusters also located on the island of Gotland (Fig. 3d).

## Spatial distribution of reliability scores

**Fig. 4:**
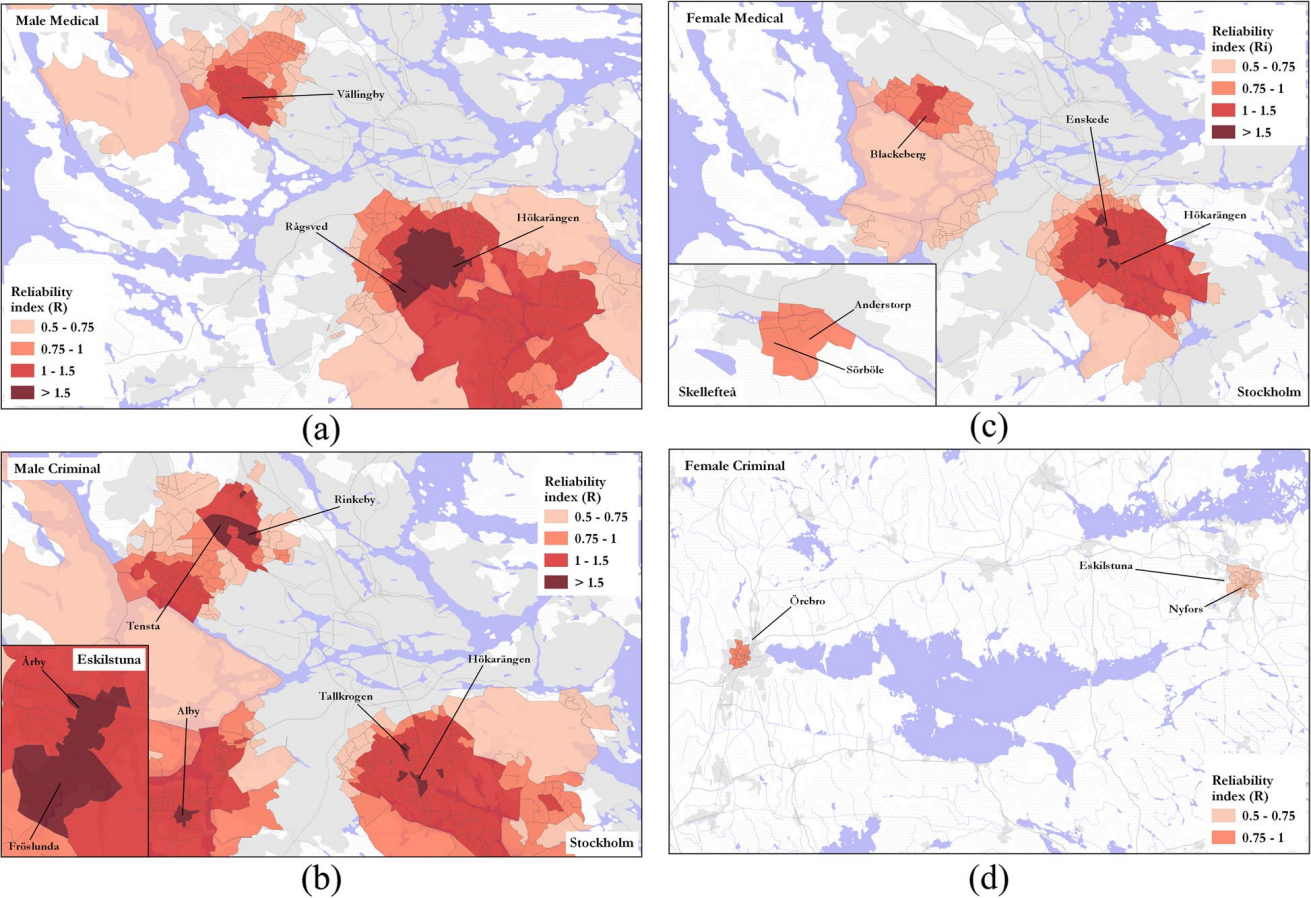
DeSOs by reliability score for gender-register categories including: **a** male–medical, **b** male–criminal, **c** female–medical and **d** female–criminal

By mapping the spatial distribution of the reliability index (*R*_*i*_), places that had a consistently high DUD rate across space and time, were identified. DeSOs with the highest male–criminal reliability scores (*R*_*i*_ > 1.5) were found in the northern and southern suburbs of Stockholm, and also in Eskilstuna (Fig. 4a), while clusters with the highest *R*_*i*_ scores for male–medical DUD rates, were also found in the northwestern and southern suburbs of Stockholm (Fig. 4b). Female-medical clusters with high reliability were also found in Stockholm’s southern suburbs, as well as in the northwest of Stockholm (Fig. 4c). Analysis of female–criminal DUD rates for the study period showed that this category had the fewest core clusters, and as such clusters with the lowest overall reliability. The most reliable areas (*R*_*i*_ > 0.75) for female–criminal register clusters were found in Eskilstuna. However, Örebro, in the southwest of the country, was also an area where *R*_*i*_ > 0.75 (Fig. 4d).

Change in local DUD prevalence across space and time.

**Fig. 5:**
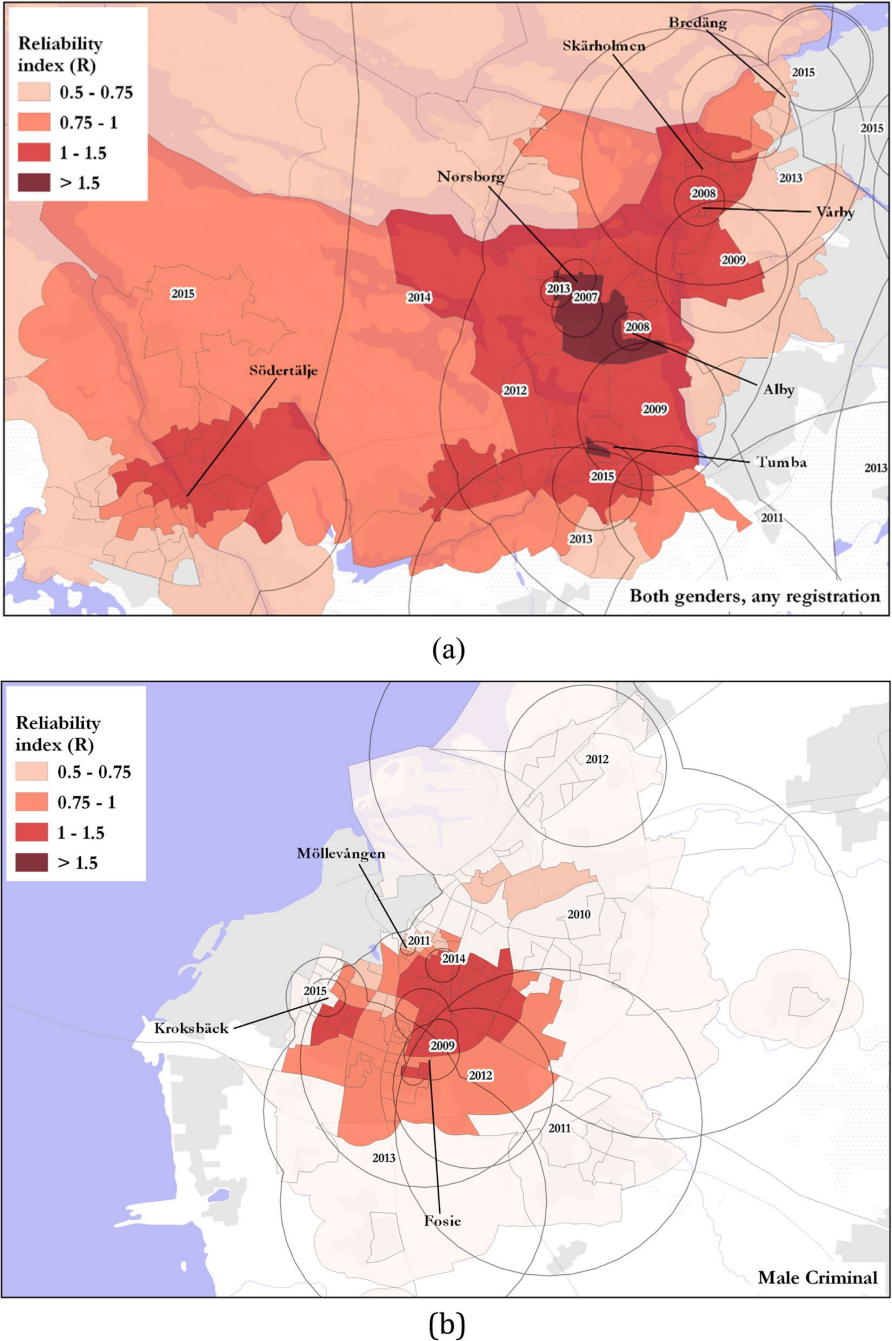
Spatio-temporal pattern of core clusters in (**a**) south-west Stockholm for all gender–register combinations and in (**b**) Malmö for male–criminal core clusters. Cluster geometries dissolved by year

The space–time clustering analysis returned results that showed numerous locations experienced a pattern where a number of early, small clusters were gradually superseded by larger expanded clusters, to be followed again in time by smaller clusters in neighboring areas. For example, in southwest Stockholm for all gender–register combinations, results returned small, early clusters in Vårby (2008), Norsborg (2007) and Alby (2008), surrounded by larger clusters later in 2009 through 2015, with localized clusters in Skärholmen and Bredäng (in 2013 and 2015 respectively) as well as Södertälje (in 2015) (Fig. 5(a)). A similar pattern was found in Malmö for male–criminal core clusters, where, for example, an early cluster in Fosie (2009) was followed by larger clusters across the eastern and northern parts of the city in the years 2010 through 2014, with later clusters in Möllevången in 2011 and Kroksbäck in 2015 (Fig. 5(b)).

In addition to having the most consistently high numbers of core clusters across all gender-registry categories, Stockholm was also notable for another persistent feature relating to DUD prevalence. A prominent gap or “hole” was consistently returned over time in the northern part of Stockholm’s city center, i.e., an area where no core cluster was reported for any gender-registry category for the study period. For male criminal registrations, the gap covers the city center (Norrmalm) in the south and extends north into Danderyd, and from west to east, it extends from Solna into a nature reserve in the East (see Fig. 7). The presence of this gap and its geographical coverage was consistent across all gender-register categories. We discuss this area further in the following section.

Controlling for neighborhood socioeconomic status.

When we controlled the model for neighborhood-level SES, the resulting overall pattern of core clusters was broadly similar. A Spearman correlation of the reliability indices across analyses controlled for SES *versus* not controlled for SES showed a relatively high correlation for medical register core clusters (correlation coefficients of 0.72 and 0.68 for males and females respectively) and slightly lower coefficient scores for core criminal clusters (0.55 and 0.41 for males and females respectively).

**Fig. 6:**
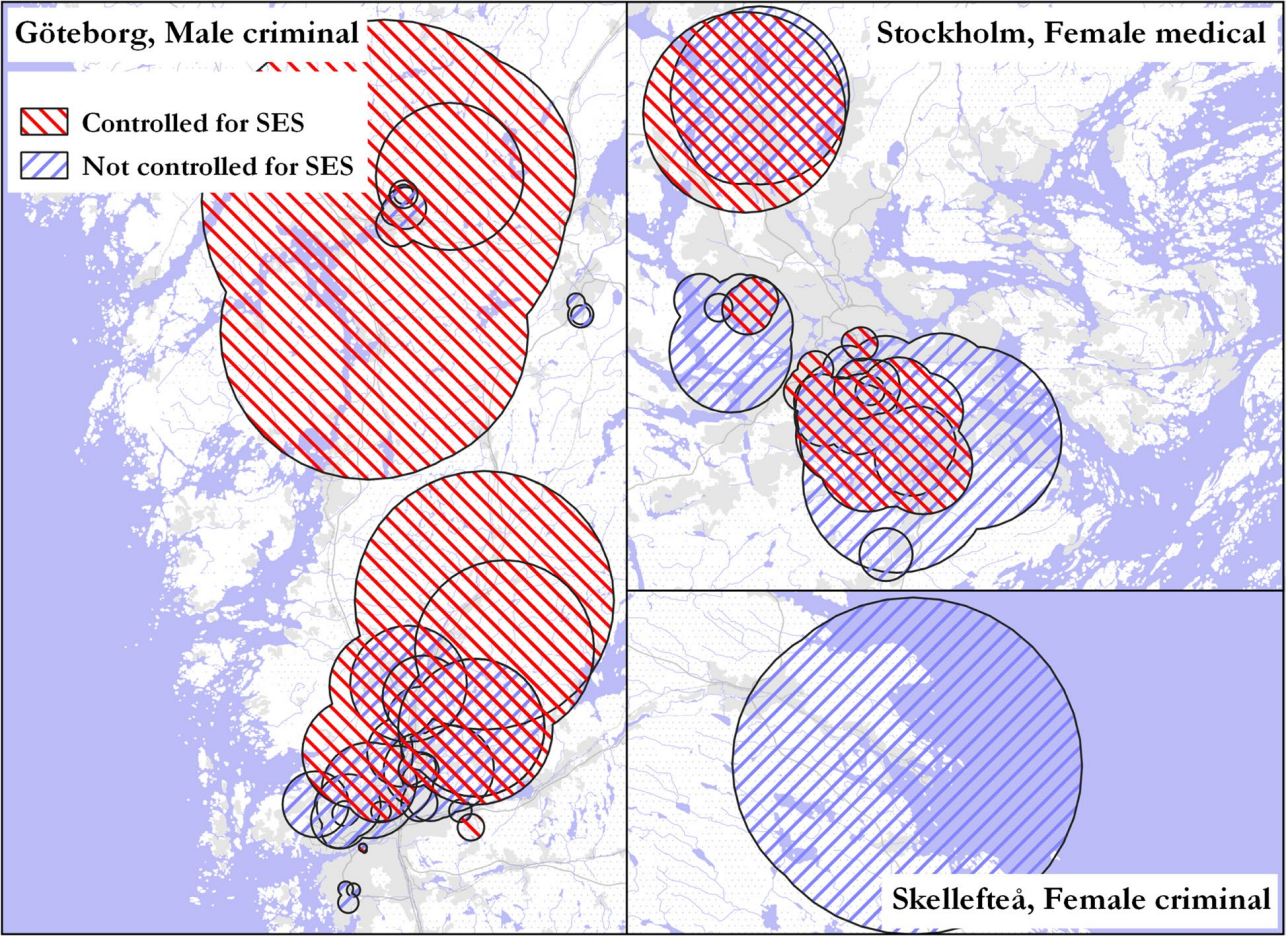
Pattern of core clusters using models that controlled for neighborhood SES and models that did not control for SES for male–criminal and female–medical clusters. Cluster geometries dissolved by year

The core clusters in models both controlled and not controlled for neighborhood SES were found to share similar locations across all gender–register categories, where the core clusters controlled for neighborhood SES were generally larger, and where the uncontrolled model identified a small number of clusters that were likely driven by neighborhood SES (Fig. 6). While the difference in sizes may be an effect of the smaller total populations across the categorical covariates resulting in larger geographies (as SaTScan tends to favor clusters at the higher end of the population threshold), these results suggest that patterns of core clusters by gender–register for 15–35-year-olds, while influenced somewhat by neighborhood SES, are not largely the result of the geographical pattern of SES itself.

**Fig. 7:**
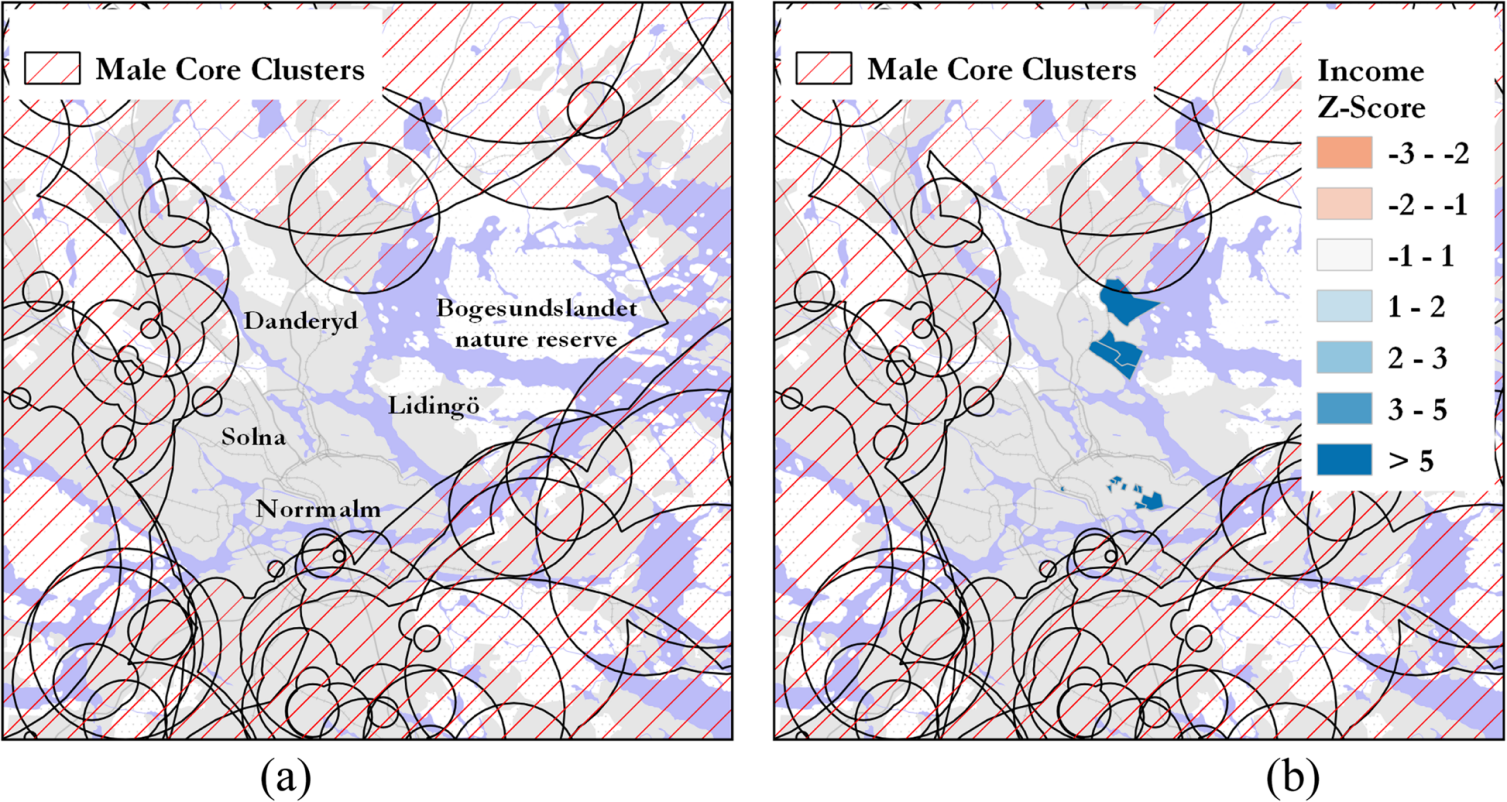
Gap in core clusters found in Stockholm for (**a**) male–criminal and male–medical core clusters and (**b**) comparison with Z-score of income for the gap region

Regarding the gap area in core clusters that persisted over time in Stockholm, there was only one DeSO area with a low SES, while the majority of DeSOs corresponded to the highest social and economic capital categories. For example, for male–criminal and male-medical clusters, no core clusters for the entire study period were returned in this area (Fig. 7a). Analysis based on mean household income for the Stockholm area shows that most of the region was well above the mean household income with some DeSOs greater than 5 S.D. above the mean income based on Z-scores (Fig. 7b). Very few DeSOs in this gap area had negative Z-scores, and among those that were negative, the presence of large student housing complexes in those DeSOs may contribute to this finding. Further analysis would be needed to understand the full set of drivers that relate to this area of consistently low DUD prevalence across all gender-registry categories.

Applying Gi* statistics to further confirm DUD case hotspots.

**Fig. 8:**
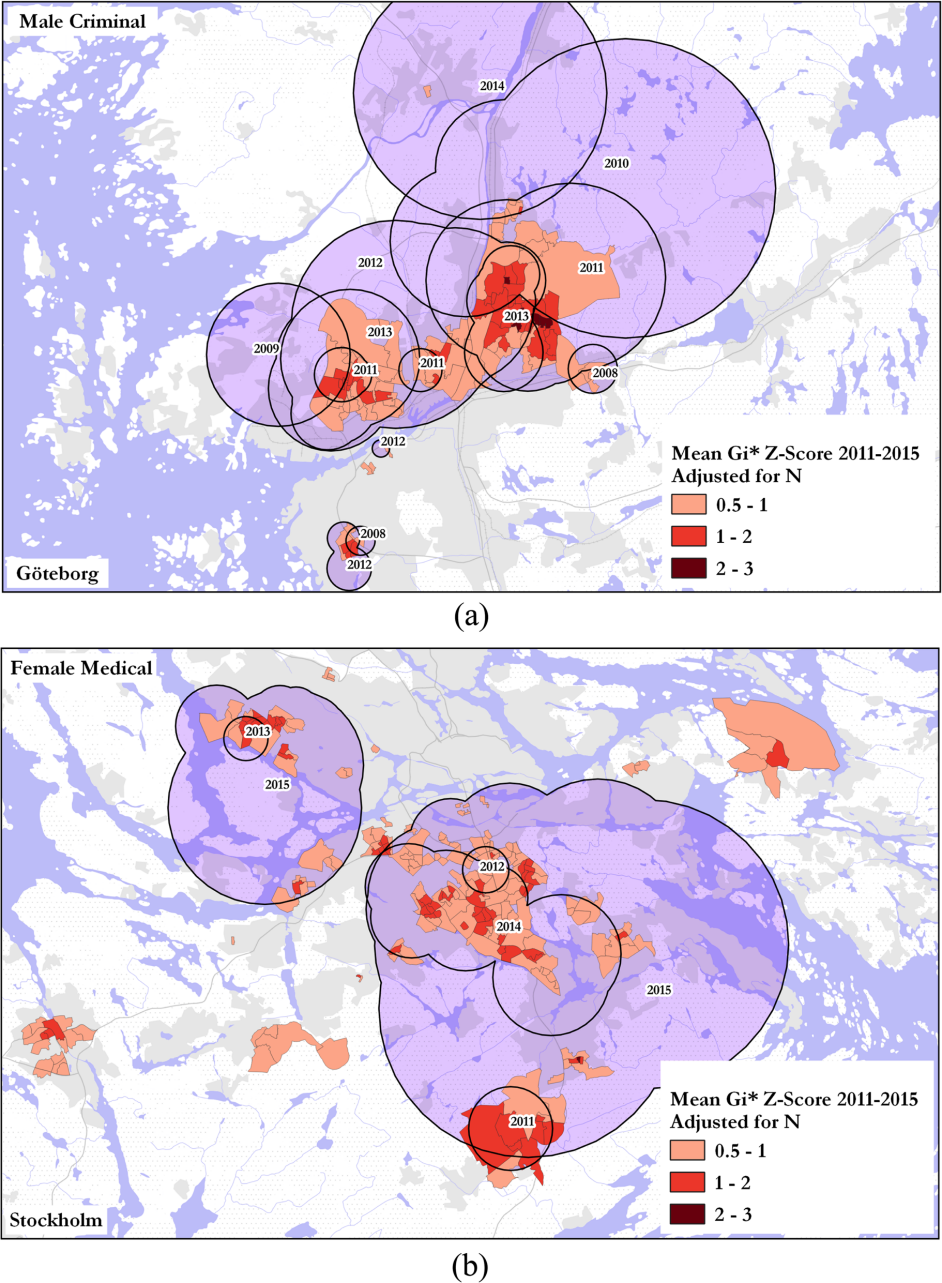
Gi* hot spot locations for (**a**) male–criminal DUD rates and (**b**) female–medical DUD and core cluster locations returned by SaTScan. Cluster geometries dissolved by year

For the last four years of the study period, 2011–2015, when core clusters for all gender-register combinations were present in numerous locations across Sweden, the spatial patterns of the mean Gi* Z-scores per DeSO were examined to understand how these results would relate to the findings of the space–time analysis. In fact, the patterns of Gi* Z-scores matched the spatial pattern of core clusters returned through the SaTScan analysis to a high degree across all gender-registry categories. In Göteborg, for example, almost all Gi* hotspots for male–criminal DUD rates with a Z-score of 0.5 or higher were located in the center of corresponding core clusters for these same years (Fig. 8a). Likewise, comparing Gi* results with female–medical core clusters in Stockholm (the largest group of female clusters), there was a similarly high degree of spatial overlap (Fig. 8b).

## Discussion

Analyzing the spatial and space–time patterns of DUD for 15–35-year-olds across Sweden between 2001–2015, our results showed, consistent with earlier evidence from a country-wide analysis of DUD-related hospitalizations [32] and from survey analyses of illicit psychoactive drug use, a general increase in prevalence over time [33]. We show, for the first time, the geospatial variations of these trends based on criminal and medical registry data unprecedented in their depth and breadth of coverage, and that has been validated in multiple prior investigations of genetic and environmental risk factors for DUD [12–16].

Our study reveals that while several cities showed higher prevalence rates of DUD for much of the study period, certain other locations emerged as hotspots only after 2011. The overall pattern saw a concentration of core clusters in and around the capital, Stockholm, while the cities of Göteborg and Malmö, in the western and southern parts of the country, respectively, were two additional locations with DeSOs with high reliability index scores throughout the entire study period. Fewer core clusters were found north of Stockholm, with the exception of the cities of Östersund in the central west region of the country and Umeå on the east coast where core clusters also were present.

For this age group, we found higher correlations in rates of DUD across medical and criminal registers than across gender. We have previously also shown high rates of cross-registration of individuals for DUD in the two registers suggesting that to a substantial, but far from complete degree, we are detecting correlated populations of drug-dependent individuals from the two registers [34]. The low correlation across gender is more interesting, pointing to relatively distinct profiles for demographic risk factors for DUD in males *versus* females, a topic warranting further investigation.

Our findings, that a higher proportion of males with DUD are detected via the criminal register and a higher proportion of affected females through the medical register is consistent with prior findings in Sweden [35]. The pathway to DUD in males more frequently involves sensation-seeking, and antisocial and criminal behaviors while females may be more likely to initially misuse psychoactive substances as a form of self-medication for symptoms of anxiety and depression [36–39]. Furthermore, as in most countries, rates of criminal offending in Sweden are substantially higher in males than females [40], further increasing the chances that DUD will be more frequently detected through criminal behavior in males than in females.

Consistent with prior studies of the concentration of DUD in urban centers in Sweden [41] and the US [42], the three largest cities in Sweden, Stockholm, Malmö, and Göteborg returned the highest number of core clusters for all gender–register combinations beginning with small, early clusters in 2008 and 2009, and then followed by larger clusters in 2010 through 2015. In each of these cities, DeSOs that comprised core clusters in 2008 and 2009 often remained part of later clusters. For the latter years, e.g., 2014 and 2015, the analysis suggested that DeSOs to the north and west of the city of Stockholm were part of an expanding set of core clusters. A persistent and noticeable gap in core clusters was found in the wealthier, northern part of Stockholm’s city center. Among those clusters that were negative, the presence of large student housing complexes in those DeSOs may contribute to this finding. This pattern also seems to fit with findings from a recent study on socioeconomic differences in drug use among adults (aged 25–64) in Sweden [14] that found that adults with higher income and education (higher socioeconomic group) reported less drug use. Nearby Eskilstuna to the west was returned as a hotspot of core clusters from 2011 on, as was the suburb of Hökarängen to the east. In the city of Malmö, by 2014, core clusters were beginning to emerge in locations north of the city while in Göteborg, the pattern was found throughout the city from 2008 on, expanding to north of Göteborg by 2014.

Our analysis of the DUD rates for 15–35-year-olds found that, consistent with a wide range of prior epidemiological findings across the world [43–45], male DUD prevalence greatly exceeded that of females although temporal trends showed a marked increase in female DUD prevalence rates over time, especially since 2012. In accord with this data, the core clusters based on criminal registrations were generally higher than medical for males, while the inverse held for females. During this period, the number of male–criminal DUD core clusters were more than 8 times higher than the number of female–criminal core clusters, and more than twice the number of female–medical core clusters. As expected, given the frequently noted negative association between SES and rates of crime, including in Sweden [41], our analyses indicated that neighborhood SES impacted the distribution of criminal DUD clusters more strongly than that of DUDs ascertained through the medical register.

Spatially, male–criminal core clusters were concentrated around the cities of Stockholm, Göteborg and Malmö, as well as in cities along the northeast coast and Östersund in the west central part of Sweden. Male–medical cluster patterns shared a similar spatial pattern, but also appeared in south central Sweden (e.g., Motala and Vadstena). Female–criminal core clusters were concentrated in DeSOs outside of Stockholm (e.g., Eskilstuna and Örebro), but not as much within Stockholm DeSOs as with the male–criminal DUD pattern. In general, female–criminal DUD had the lowest prevalence and was the category with the most noise. Locations on the west coast of Sweden that were present for both male–criminal and male–medical DUD, were not found for females and neither were any locations in the north returned as core clusters for females. Female–medical core clusters, however, did appear in Stockholm and its suburbs, as well as in the cities of Eskilstuna, and Göteborg, in addition to the island of Gotland.

It should be noted that while DeSO areas are designed with spatial boundaries in mind, the SaTScan model does not take boundary effects into account. This can lead to areas that in reality may have little interaction, being aggregated into the same cluster [46, 47]. Although we do not believe this is a major concern, we can see some effect of this especially in the Stockholm area, where a small number of clusters cut across water bodies and connect disparate neighborhoods. Recent studies have investigated scan statistics for irregular clusters [48]. Another possible limitation is that for this spatial analysis, only individuals that could be connected to a DeSO for the relevant year were included in the population total. This means there may be a minor underestimation of the total population count for the age group being studied. Also, it should be noted that each individual has been assigned to a DeSO via their registered place of residence according to Skatteverket, the Swedish Tax Agency. There may be some discrepancies between where people are registered as residents and where they actually live, which may contribute uncertainty in the neighborhood-based studies. There could also be some uncertainty related to the reliability of place of residence information based on local errors that are due to incorrect registrations of place of residence, but this error is likely to be rare. We could not incorporate into our models the rise in drug testing of criminals that has occurred in Sweden over our time period of interest [49]. This could have has contributed to some of the observed overall rise in DUD rates in the criminal registry but cannot explain the parallel increases seen in the medical registry. The SES index used to control for socioeconomic status was originally designed to identify neighborhood deprivation specifically rather than continuous SES. As such, the distribution of the index per DeSO may be skewed. This may mask a larger impact of neighborhood socioeconomic gradient than we have seen in this study. Finally, it is possible that estimates for DUD prevalence from some populations covered by the Swedish National Patient Registry, for example, those coming from psychiatric outpatient care in Stockholm, especially for the early years of this study (e.g., between 2001–2006) may be underestimations. If this is the case, this could lead to a possible downward bias in the number of clusters in the Stockholm area for those years. Further study would be necessary to determine how the time-varying patterns of spatial clusters are impacted from such differences in the completeness of DUD ascertainment.

## Conclusions

This study has investigated space–time patterns of DUD across Sweden between 2001 and 2015 at the spatial granularity of DeSOs for a key age group, 15–35, when the first onset of DUD is most likely to be experienced. We used data from the high quality medical and criminal registers available in Sweden, stratifying our space–time results by both gender and the type of register. Prevalence was measured as the ratio of individuals with at least one DUD-registration in each register by the population per geographic unit during each year. The analysis focused on core clusters that represented groupings of DeSOs filtered based on relative risk as well as a further statistical test in order to identify key hotspots of DUD prevalence. Applying space–time clustering using SaTScan revealed three persistent hotspots in Stockholm, Göteborg and Malmö, confirmed separately using a Gi* analysis. By the end of the study period, each of these hotspots were showing further expansion into DeSOs in other nearby towns and cities. Male DUD prevalence greatly exceeded that of females although temporal trends showed a marked increase in female DUD prevalence rates over time, especially since 2012. We found higher correlations in rates of DUD between medical and criminal registers than across gender with a higher proportion of males with DUD detected via the criminal register and a higher proportion of females with DUD through the medical register. Male–criminal core clusters were concentrated in the cities of Stockholm, Göteborg and Malmö, as well as in cities along the northeast coast and in Östersund. Female–medical core clusters appeared in Stockholm and its suburbs, as well as in the nearby cities of Eskilstuna, and Göteborg, and the island of Gotland. Male–medical cluster patterns shared a similar spatial pattern to that of male–criminal, but also appeared in cities such as Motala in south central Sweden. The findings are important for identifying drivers that underlie the spatial patterns of spread, for monitoring the current drug epidemic, and for generating hypotheses about drug prevalence in local areas.

We plan in the future to use these results as a geographic framework to answer a range of questions about the etiology of DUD in Sweden that have previously been examined by standard regression analysis in all of Sweden. Such questions will include an in-depth analysis of effects of migration into and out from these newly identified high-risk areas and modifying effects of social and familial factors. Questions we hope to address would include to what extent do these hotspots arise from high rates of first onsets of DUD for resident individuals versus in-migration of already affected individuals, and what is the risk for DUD associated with migration into or out of these high-risk areas and are these results consistent across hotspots. An analysis of what social and familial factors make residents in these hotspots especially vulnerable to DUD and whether these differ from the risk factors that operate in low-risk regions would also be a valuable topic to pursue.

## Data Availability

The data used in this study is not readily available from the researchers, who were granted permission to use these data from the Swedish authorities. Due to restrictions regulated by law, they are not allowed to share the data. However, researchers who wish to use these data could contact Kristina Sundquist for further information and guidance.

## Abbreviations

DeSO: Demographic Statistical Area
DUD: Drug Use Disorder
Gi*: Getis-Ord Statistic
Ri: Reliability index
Rr: Relative risk
SAMS: Small Areas for Market Statistics
SD: Standard deviation
SES: Socioeconomic status
